# EDITOR’S MESSAGE / MESSAGE DE LA RÉDACTION

**DOI:** 10.29173/jchla29803

**Published:** 2024-08-01

**Authors:** Megan Kennedy

**Affiliations:** JCHLA/JABSC Editor

[Français en suite]

Welcome to the August 2024 issue of JCHLA/JABSC! I hope everyone is enjoying the summer weather and taking some time to rest and relax. I am very pleased to present this issue which contains a special section on literature related to health librarianship and libraries during the COVID-19 pandemic. This was an extraordinary time to work in any health-related field, and health libraries were no exception. We have several program descriptions that showcase the innovative work undertaken by health librarians during this time. Additionally, we have a column from the librarians and library technicians of Saskatchewan Health Authority Library who won the CHLA/ABSC Flower Award for Innovation in 2022 for their work during the COVID-19 pandemic.

This issue also includes two book reviews on post-pandemic libraries and accreditation in the health sciences and a product review of the Scopus search analyzer.

I was fortunate to be able to attend the wonderful CHLA/ABSC conference in beautiful Winnipeg in June. It was great to see colleagues from across the country come together and talk about all things health librarianship - we are certainly a small but mighty community. Abstracts for the conference's contributed papers, lightning talks, and posters can be found in this issue.

This issue marks the end of my three-year term on the JCHLA/JABSC Editorial Board. This experience has been very rewarding for me and I am so thankful to the JCHLA/JABSC and CHLA/ABSC members who have provided their time and mentorship over the years. Starting in September, Jessica McEwan (University of Ottawa) will take on the role of Editor, and Ashley Farrell (University Health Network) will move into the position of Senior Editor. Tyler Ostapyk (University of Manitoba) will continue as Production Editor. We are very pleased to welcome two new members to the Editorial Board; Melissa Walter (CADTH) as Junior Editor and Kate Merucci (Health Canada) as Copyeditor. We thank our outgoing Copyeditor, Meredith Fischer (Wilfred Laurier University), for all her hard work over the past two years.

As always, thank you to all our authors and peer reviewers for your contributions and continued support.

**Megan Kennedy**

*JCHLA/JABSC Editor*

*Email: editor@chla-absc.ca*

Bienvenue au numéro d'août 2024 du JABSC/JCHLA! J'espère que tout le monde profite de la température estivale et prend le temps de se reposer et de se détendre. Je suis très heureuse de vous présenter ce numéro qui contient une section spéciale sur la littérature relative à la bibliothéconomie et aux bibliothèques de santé pendant la pandémie de la COVID-19. Ce fut une période extraordinaire pour travailler dans tous les domaines liés à la santé, et les bibliothèques de santé n'ont pas fait exception. Nous avons plusieurs descriptions de programmes qui illustrent le travail innovant entrepris par les bibliothécaires de santé pendant cette période. En outre, nous avons une chronique rédigée par des bibliothécaires et des techniciens et techniciennes de bibliothèque de la Saskatchewan Health Authority Library qui ont remporté le ABSC/CHLA Flower Award for Innovation en 2022 pour leur travail pendant la pandémie de la COVID-19.

Ce numéro comprend également deux critiques de livres sur les bibliothèques post-pandémiques et sur l'agrément dans les sciences de la santé, ainsi qu'une évaluation de produit portant sur l'analyseur de recherche Scopus.

J'ai eu la chance de pouvoir assister à la merveilleuse conférence de l'ABSC/CHLA qui s'est tenue dans la magnifique ville de Winnipeg en juin. C'était formidable de voir des collègues de tous les coins du pays se réunir et parler de tout ce qui touche à la bibliothéconomie de la santé - nous sommes une petite mais très vigoureuse communauté. Vous trouverez dans ce numéro les résumés des communications, des conférences éclair et des affiches de la conférence.

Ce numéro marque la fin de mon mandat de trois ans au sein du comité de rédaction du JABSC/JCHLA. Cette expérience a été très enrichissante pour moi et je suis très reconnaissante aux membres du JABSC/JCHLA et de l’ABSC/CHLA qui ont offert leurtemps et leur mentorat au fil des ans. À partir de septembre, Jessica McEwan (Université d'Ottawa) assumera le rôle de rédactrice en chef, et Ashley Farrell (University Health Network) deviendra rédactrice principale. Tyler Ostapyk (Université du Manitoba) restera rédacteur de laproduction. Nous sommes très heureux d'accueillir deux nouvelles membres au sein du comité de rédaction : Melissa Walter (ACMTS) en tant que rédactrice débutante et Kate Merucci (Santé Canada) en tant que réviseure . Nous remercions notre réviseure sortante, Meredith Fischer (Wilfred Laurier University), pour son travail acharné au cours des deux dernières années.

Comme toujours, nous remercions tous nos auteurs et auteures et nos pairs évaluateurs et évaluatrices pour leurs contributions et leur collaboration soutenue.

**Megan Kennedy**

*Rédactrice en chef du JCHLA/JABSC*

*Courriel: editor@chla-absc.ca*

